# Guaiacol in Phthisis

**Published:** 1898-04-16

**Authors:** 


### GUAIACOL IN PHTHISIS.

Dr. Edward Squire has been giving guaiacol in considerable doses to patients at the North London Hospital for Consumption, and his results are rather interesting, showing, as they apparently do, that this drug, at any rate, when given in conjunction with the general hygienic treatment of a well-conducted hospital, has some influence in controlling the progress of the disease. The drug was given in somewhat large doses; six cases took 60 minims, two cases took 50 minims, four cases took 40 minims, six cases took 30 minims, ten cases took 20 minims, six cases took 15 minims, and six cases took 10 minims three times a day after their meals. The guaiacol was at first administered in capsules, containing five minims each, but afterwards in an emulsion with glycerine and tincture of orange peel. Only one patient felt any ill effect. He was taking 20 minims three times a day when he complained of much stomachic and abdominal pain, with a sinking sensation at the epigastrium, and passed a small quantity of blood by the bowel. The guaiacol was stopped, but resumed later in 10 minim doses without ill-effect. Twenty-six of the patients were cavitation cases, and in these the diminution of the amount of expectoration was very marked, the diminution beginning early in the administration of the drug. The cough did not seem to be materially affected, but the night sweats in nearly all cases diminished, and then disappeared in a very short space of time. The explanations which may be offered of the action of drugs in phthisis are various, and are often tinged with scepticism. Fortunately, it is permissible to use a remedy which experience has shown to do good without waiting to know exactly how it acts. Many of the patients complained of the guaiacol producing a sense of burning in the throat, but this difficulty was much lessened by drinking half a cupful of milk immediately afterwards.

